# Catheter directed hepatic artery thrombolysis following liver transplantation. Case report and review of the literature

**DOI:** 10.1259/bjrcr.20190005

**Published:** 2019-04-29

**Authors:** Miguel Ángel Carrillo-Martínez, Carlos Rodríguez-Montalvo, Eduardo Flores-Villaba, Lucas Tijerina-Gómez, Francisco Edgardo Puente-Gallegos, Samuel Eugene Kettenhofen, German Alfonso Garza-García

**Affiliations:** 1 Department of Interventional Radiology, Tecnologico de Monterrey, Escuela de Medicina y Ciencias de la Salud, Mexico; 2 Deparment of Surgery, Tecnologico de Monterrey, Escuela de Medicina y Ciencias de la Salud, Mexico; 3 Department of Diagnostic Radiology, Tecnologico de Monterrey, Escuela de Medicina y Ciencias de la Salud, Mexico; 4 Tecnologico de Monterrey, Escuela de Medicina y Ciencias de la Salud, Mexico

## Abstract

Hepatic artery thrombosis is the most frequent vascular complication following orthotopic liver transplantation, and often results in biliary complications, early graft loss and death. Surgical revascularization and retransplantation are considered the mainstay of treatment. However, intraarterial endovascular therapy is an alternative that has shown low morbidity and mortality, thereby avoiding the need for retransplantation. We describe a case of orthotopic liver transplantation complicated with hepatic artery thrombosis that was successfully treated with endovascular therapy.

## Background

Vascular complications following orthotopic liver transplantation have a direct influence on graft viability and patient mortality. Post-operative complications include hemorrhage, stenosis, and thrombosis at any vascular anastomosis, however, hepatic artery thrombosis (HAT) and portal vein thrombosis (PVT) are considered the most common complications.^1^

HAT and PVT interrupt adequate graft vascularization which leads to graft dysfunction, loss and even patient death, making this a serious complication requiring urgent revascularization.

Surgical revascularization and urgent retransplantation are considered as the mainstay therapy, however endovascular therapy is an attractive alternative with a comparatively high success rate and low mortality and morbidity.

## Clinical Presentation

A 39 year-old male with a past medical history of sclerosing cholangitis and hepatic cirrhosis classified as Child-Pugh C was subjected to a cadaveric whole liver transplantation. Conventional surgical technique was performed with resection of the inferior vena cava and venovenous bypass with a total cold ischemic time of 460 min. Post-operative immunosuppression therapy included tacrolimus, mycophenolate mofetil, and steroids. Anticoagulant therapy was not administered.

A Doppler ultrasound on post-transplant Day 2 (PTD) demonstrated absence of flow in the hepatic artery. An emergent exploratory laparotomy attributed this finding to thrombosis at the site of vascular anastomosis and an aorto-hepatic bypass was placed with a No. six ringed GORE-TEX ^®^ vascular graft (W.L. Gore & Associates, Flagstaff, AZ).

**Figure 1.**
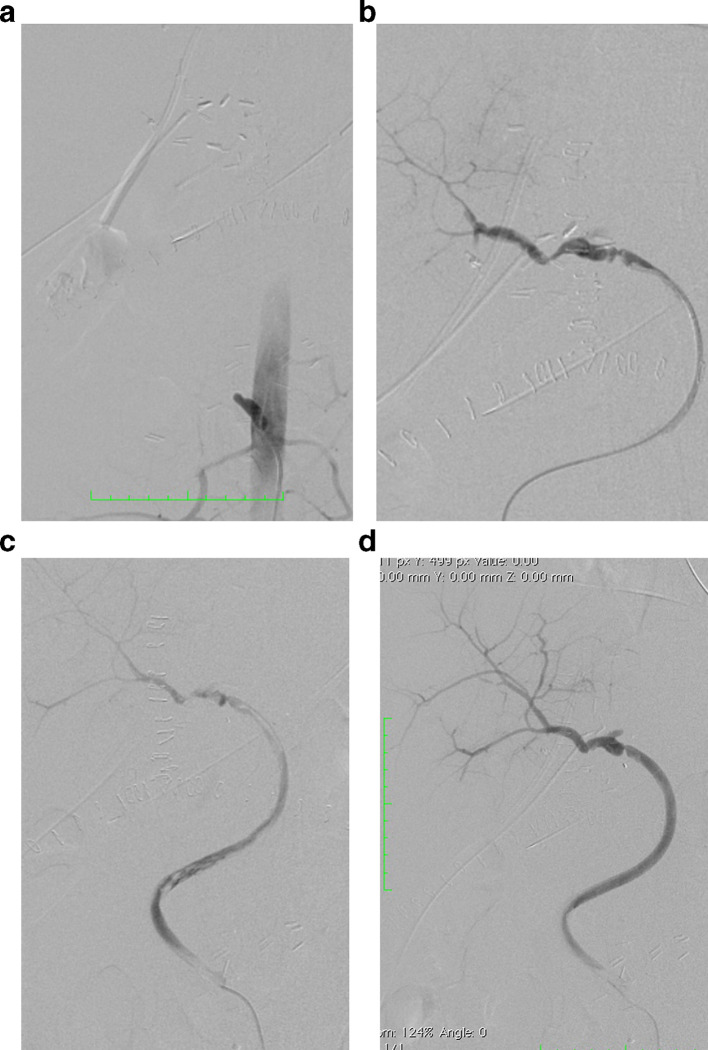
(a) Angiography showing occlusion of theaorto-hepatic vascular graft. (b) Guidewire and catheter placement inoccluded vascular graft. (c) Catheter-directed thrombolysis of the vascular graft with rTPA. (d) Control angiography after continuous infusion of rTPA shows revascularization of the right hepatic artery while occlusion of the left hepatic artery persists.

**Figure 2.**
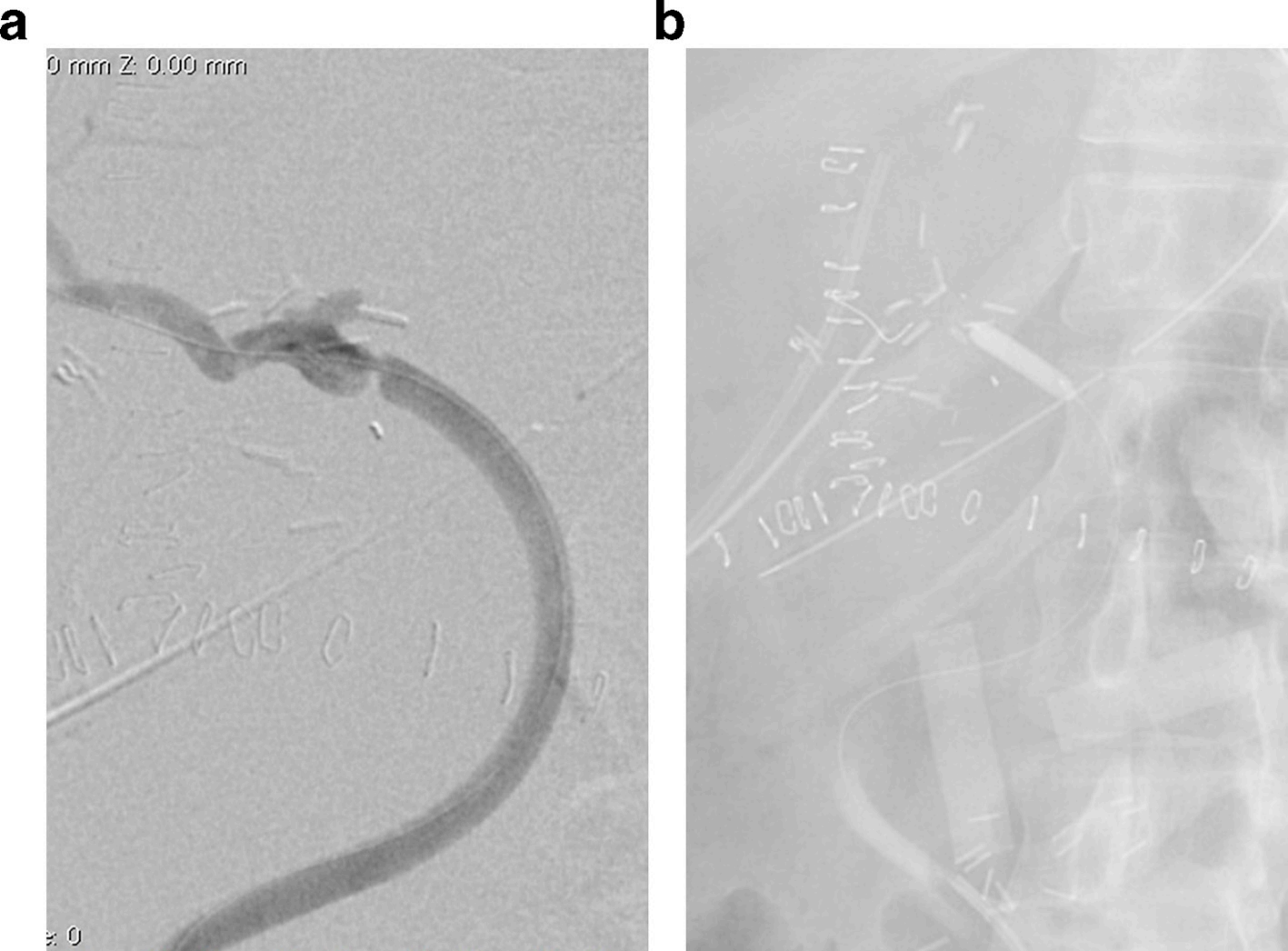
(a) Control angiography post-thrombolysisdemonstrating stenosis at the site of anastomosis of the vascular graft withthe hepatic artery. (b) Balloon dilatation of stenosis with a 5x20 mm balloon.

**Figure 3.**
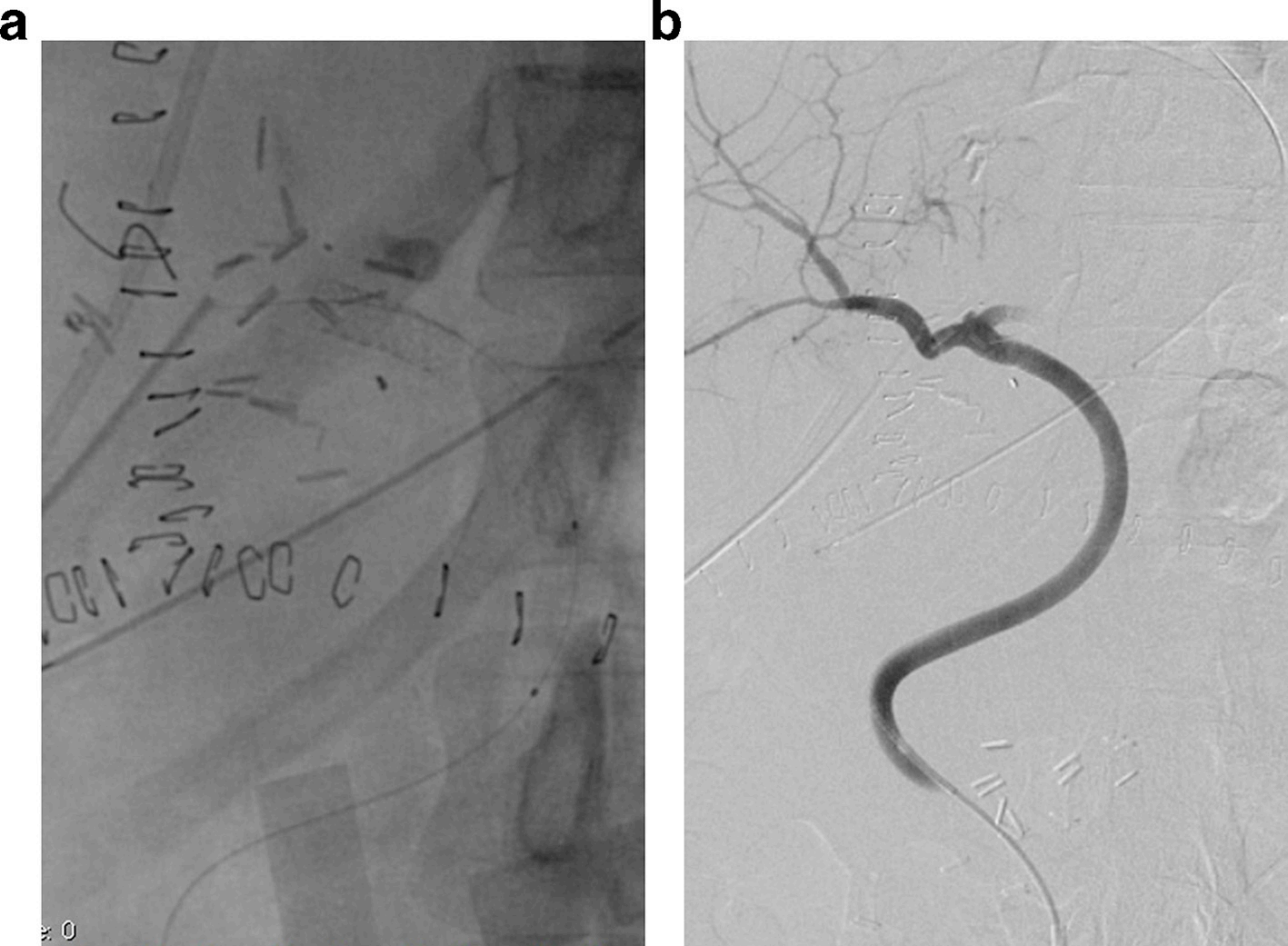
Hipocampus® stent before (a) and after (b) deployment at the site of stenosis in the vascular graft with revascularization of the vascular graft and right-hepatic artery.

On PTD three the patient developed hyperbilirubinemia secondary to an increase in the direct bilirubin (total bilirubin: 6.1 mg dl^−1^, direct bilirubin: 4.74 mg dl^−1^) with increased liver enzymes (aspartate transaminase: 172 U l^−1^, alanine transaminase: 70 U l^−1^ and alkaline phosphatase 414 U l^−1^). A hepatic ultrasound reported absence of flow in the hepatic artery at the hilum without evidence of intrahepatic arterial flow. Occlusion of the aortohepatic vascular graft was confirmed with angiography (Figure 1a). Although there was no contraindication for surgical revascularization, endovascular management was elected as an initial therapeutic option considering our teams experience on a previous case, reserving surgery for technical failures or complications. A Teflon^®^ coated guidewire (Cordis, Santa Clara, CA) and a 4 Fr catheter were used to access the graft and perform catheter-directed thrombolysis with recombinant tissue plasminogen activator (rtPA) 4 mg initial dose followed by 4 mg continuous infusion for 20 min (Figure 1b,c,d). Post-thrombolysis control angiography demonstrated significant stenosis at the hepatic anastomosis of the graft and balloon dilatation was performed with 5 × 20 mm and 6 × 20 mm balloons with unfavorable results due to immediate elastic restenosis (Figure 2a,b). Subsequently, a 6 × 20 mm Hippocampus^®^ stent (Medtronic, Minneapolis, MN) was placed, achieving adequate flow in the vascular graft and the main branches of the right hepatic artery. (Figure 3a,b). Proximal occlusion of the left hepatic artery with distal collateral circulation was observed.

**Figure 4.**
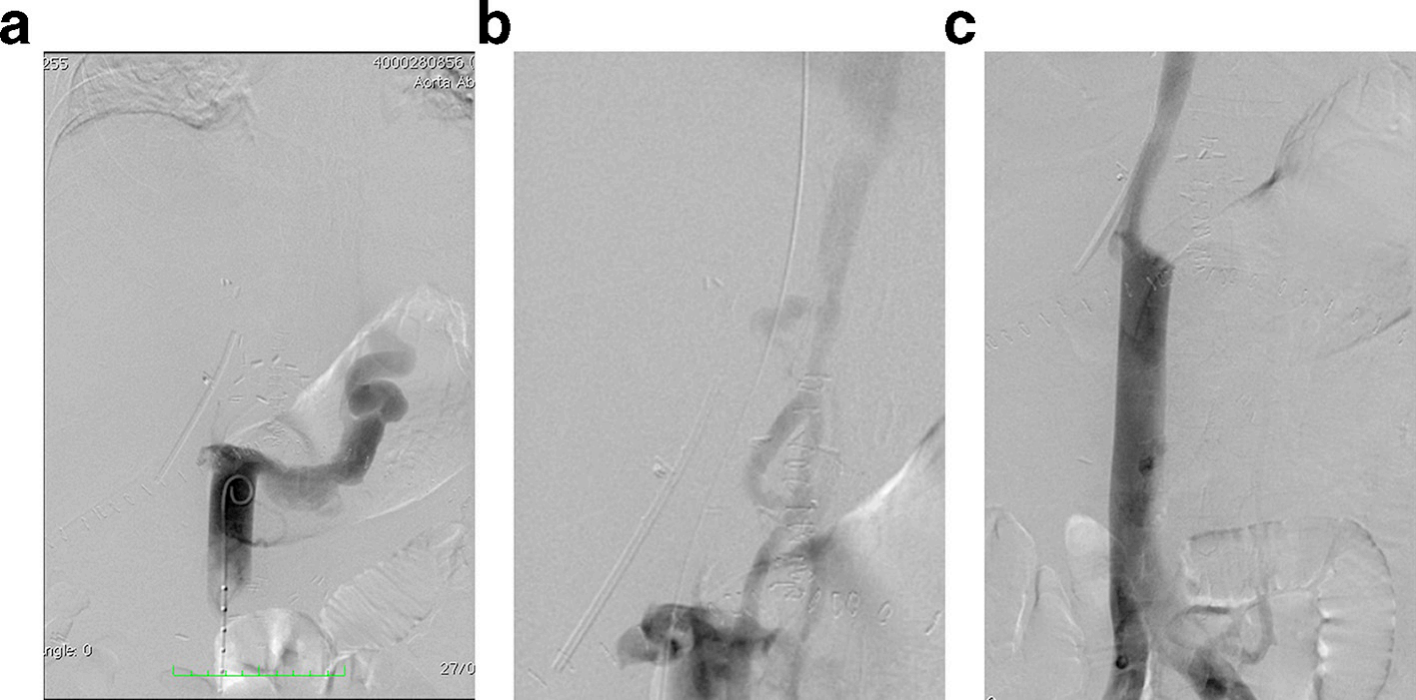
Cavography demonstrating occlusion of the IVC at the level of the renal veins. Collateral renal vein (a) and azygos (b) circulation. (c) Controlvenography shows revascularization with adequate flow towards the right atrium.

**Figure 5.**
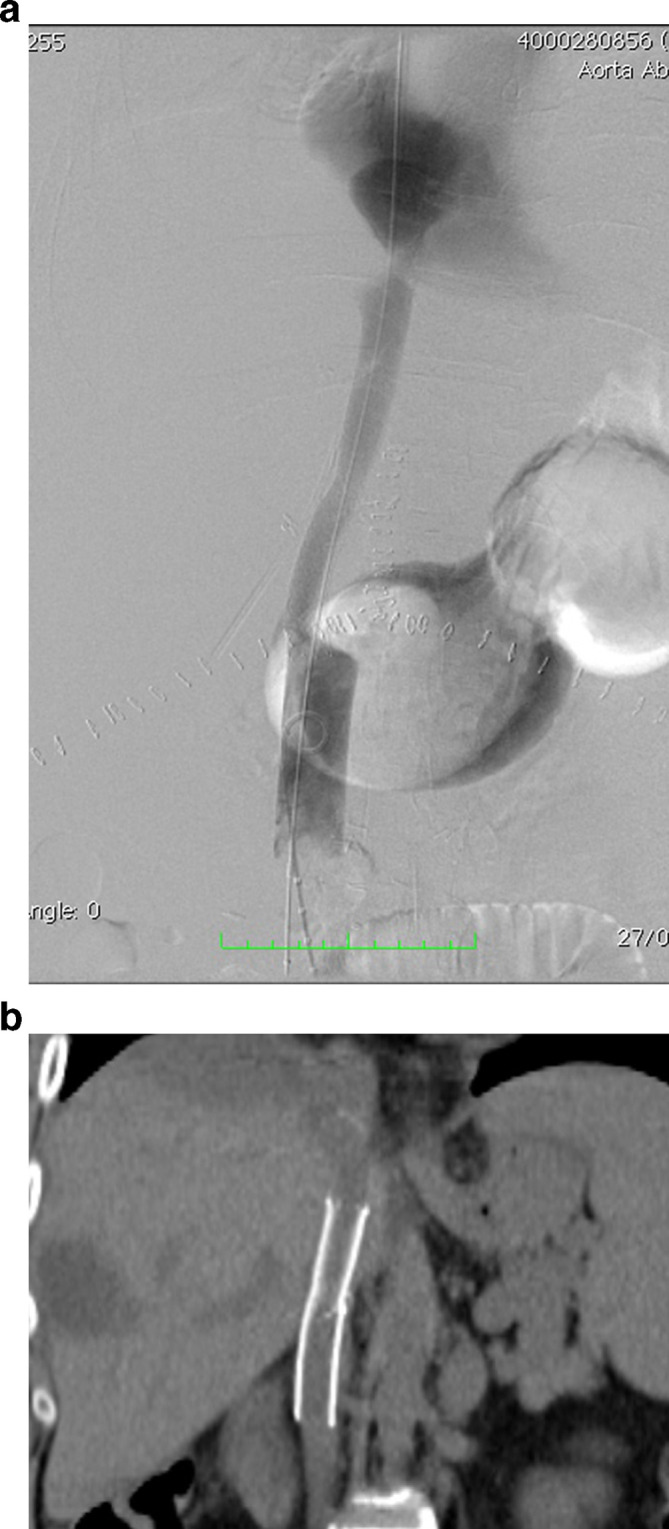
(a) Controlvenography shows revascularization with adequate flow towards the right atrium. (b) Abdomen CT with coronal reconstruction shows stent placement in the IVC.

The patient’s clinical condition deteriorated on PTD 14, presenting with lower extremity edema, dyspnea during rest, tachycardia, tachypnea and hypoxemia (89% pulse oximetry). Thrombosis of the inferior vena cava (IVC) was suspected and a cavography was performed, confirming occlusion of the infrarenal IVC with collateral filling (Figure 4a,b). A multipurpose catheter was inserted and a guidewire was used to recanalize the IVC. Pre-dilatation with a 10 mm balloon catheter was performed and a 14 × 80 mm self-expanding S.M.A.R.T.^®^ stent (Cordis, Santa Clara, CA) was placed and expanded to 14 mm (stents of a greater caliber were unavailable) (Figure 4c). Control cavography demonstrated adequate recanalization of the IVC with anterograde flow and absence of collateral filling (Figure 5a,b). The patient had a favorable evolution and was discharged approximately 1 month post-transplant.

3 months after discharge, the patient developed an abscess at a previously detected site of focal hepatic necrosis, requiring percutaneous drainage with complete resolution after 3 weeks.

## Discussion

HAT is the most common and severe vascular complication following orthotopic hepatic transplantation, accounting for approximately 50% of vascular complications and with reported mortality as high as 75%.^2,3^ It is defined as a thrombotic occlusion of the hepatic artery and is classified as early or late depending on time of presentation following liver transplantation. Early HAT occurs within 30 days and is associated with a rapid clinical deterioration, a high rate of graft loss and increased mortality rates. Late HAT occurs after 30 days post-transplantation and has a relatively mild clinical course.^4^ Because the hepatic artery is the only route by which oxygenated blood is supplied to the liver, its obstruction results in parenchymal and biliary ischemia. Clinically, its presentation varies from slight signs of parenchymal involvement to fulminating hepatic failure.

A determining factor that limits hepatic injury and graft loss is the early detection of arterial complications by means of Doppler ultrasound of the liver and angiography.^3^ Clinical markers that may indicate early HAT include fever, leukocytosis, elevation of hepatic enzymes, hiperbilirubinemia, and shock. Other patterns that may also suggest HAT include fulminant hepatic failure, late-onset biliary leakage, and recurrent bacteremia.^5^ Doppler ultrasound within the first 3 days post-transplantation provides the advantage of diagnosis of HAT before clinical manifestations become apparent.^3^

Three treatment options have been described for HAT: surgical revascularization, urgent retransplantation and endovascular revascularization. Retransplation is considered the first-line therapy, however, it often carries a higher mortality rate and is limited by a shortage of donors.^6^ Urgent endovascular revascularization therefore plays an important role in the management of HAT, potentially avoiding the need for retransplantation in cases where a diagnosis is made before signs and symptoms of graft dysfunction become apparent.^2^ Sheiner et al report a revascularization survival rate of 40 and 82% in symptomatic and asymptomatic patients, respectively.^7^

Intra-arterial thrombolysis for HAT was first described by Hidalgo *et al* in 1989.^8^ It is thought that thrombolysis is most effective in “fresh” thrombi due to a higher water content and a matrix relatively poor in fibrin. Literature on this particular topic varies greatly, some recommending thrombolysis as early as 4 h and others as late as 120 days post-transplantation. There are no current guidelines regarding the therapeutic window for thrombolysis. Nevertheless, the general consensus is that it should not be attempted more than 3 months post-transplantation.^2^

Hepatic-artery thrombolysis is considered a safe treatment, with the most common and severe complication being hemorrhage reported in approximately 20% of patients. The main pitfall of thrombolysis is short-term recurrence of HAT if the conditions that favored its formation are not resolved (*e.g.* kinks, twist, dissection or stenosis at the site of anastomosis). Angioplasty and stent placement after intraarterial thrombolysis have become a solution with more favorable results.^9,10^ It is important to mention that multiple points of stenosis as well as HAT in pediatric patients are not considered absolute contraindications for thrombolysis and angioplasty, particularly if retransplantation is not an option.

Our patient also presented with thrombosis of the IVC, a rare complication estimated to occur in 1–2% of transplants.^11^ It is most often related to surgical technique and generally occurs within the first month after transplantation. Without prompt diagnosis and therapeutic intervention, it is associated with high morbidity, mortality and graft failure. There are several reports of successful management of thrombosis of the IVC with thrombolysis and stent placement.^12^ Surgical repair may be unavoidable in cases of restenosis or kinking of the graft due to size mismatch between the donor and recipient.

## Conclusion

HAT is a serious complication of liver transplantation and early detection is crucial to preserve function and viability of the graft and improve patient survival. Post-operative hepatic Doppler ultrasound is an important tool in early diagnosis, although angiography is currently the gold standard in the detection of this complication with the added benefit of permitting endovascular therapeutic options. Catheter directed thrombolysis is safe and effective, although restenosis is common if the predisposing factor is not corrected. However, when combined with post-thrombolysis balloon angioplasty and stent placement, therapeutic results and patient outcome may improve. Although the ideal treatment of symptomatic patients and those with severe graft dysfunction is urgent retransplantation, the shortage of donor livers makes endovascular therapy an attractive therapeutic option to save the graft and avoid retransplantation or facilitate it in an elective setting.

## Learning points

- Early detection of hepatic artery thrombosis is critical to conserve graft vitality.
- Doppler ultrasound assessment must be done by experienced sonographers and in case of doubt, an angiography or angiotomography must be performed.
- Thrombolysis of the hepatic artery is safe and effective.
- After thrombolysis, balloon angioplasty with or without stent placement may be needed.
